# Comparison of Patellar Tracking Following Kinematic Alignment Versus Mechanical Alignment Total Knee Arthroplasty via the Mini‐Subvastus Approach

**DOI:** 10.1111/os.70016

**Published:** 2025-03-10

**Authors:** Bochen Sun, Yiyang Xu, Guiguan Wang, Long Chen, Fenqi Luo, Guoyu Yu, Yuan Lin, Jie Xu

**Affiliations:** ^1^ Department of Orthopedics, Fujian Provincial Hospital, Shengli Clinical Medical College Fujian Medical University, Fuzhou University Affiliated Provincial Hospital Fuzhou China; ^2^ Department of Orthopedics Yun Xiao County Hospital Zhangzhou China

**Keywords:** kinematic alignment, mechanical alignment, mini‐subvastus approach, patellar tracking, total knee arthroplasty

## Abstract

**Objectives:**

Different alignment strategies (kinematic alignment [KA] versus mechanical alignment [MA]) during total knee arthroplasty (TKA) significantly influence postoperative patellar tracking. This study aimed to compare radiological parameters of patellar tracking and clinical outcomes between KA‐TKA and MA‐TKA via the mini‐subvastus approach.

**Methods:**

This prospective randomized controlled study included 234 patients who underwent KA‐TKA and MA‐TKA from January 2022 to October 2023. The preoperative and postoperative patellar tilt, lateral patellar shift, knee society score (KSS), oxford knee score (OKS), and intraoperative patellar lateral retinacular release (LRR) rate were measured. In addition, radiological parameters and clinical outcomes were compared between the LRR and non‐LRR groups. Independent samples *t* test and chi‐square test were used to compare the differences between groups.

**Results:**

Two‐hundred and thirty‐four patients were followed up for 12 months post‐TKA. No significant differences were observed between the two groups in terms of the demographics and pre‐ or post‐operative radiological parameters of patellar tracking (*p* > 0.05). The postoperative KSS and OKS were significantly higher in the KA group than in the MA group (*p* < 0.05). The LRR rate was 6.7% (8/120) in the KA group and 25.4% (29/114) in the MA group, and the difference was statistically significant (*x* ^2^ = 15.476, *p* < 0.001). The preoperative patella tilt and lateral patellar shift were greater in the LRR group (*p* < 0.001) and the postoperative OKS was lower (*p* < 0.05).

**Conclusions:**

KA‐TKA via the mini‐subvastus approach can achieve both good patellar tracking and clinical outcomes. Avoiding muscle damage and refraining from excessive soft tissue release are crucial to improving postoperative patient comfort. In our opinion, KA‐TKA via the mini‐subvastus approach may be a more suitable surgical option.

## Graphical abstract

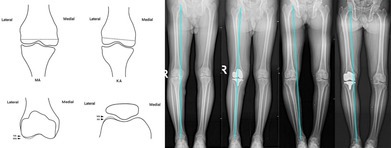

## 1 Introduction

Osteoarthritis of the knee is the most common disease in orthopedics, and total knee arthroplasty (TKA) is the mainstay of treatment for end‐stage knee osteoarthritis [1]. However, there are complications after TKA, such as anterior knee pain, wound infection, deep vein thrombosis, patellar fracture, osteolysis, and prosthesis loosening. Among these outcomes of TKA, anterior knee pain is the most common [2] and is related to changes in inherent joint anatomy and soft tissue tension after TKA surgery that impact patellar tracking.

Most knee surgeons consider mechanical alignment (MA) to be the gold standard for TKA because it is associated with longer survival. However, Bellemans et al. [3] reported that the knee has constitutional varus in the normal population and that MA‐TKA pursues a neutral alignment that is not suitable for all patients. Kinematic alignment (KA) is a personalized surgical technique that employs equal osteotomy with hardly any soft tissue release and aims to maximally restore the patient's knee joint to its native state [4]. Currently, most studies on patellar tracking following KA‐TKA are based on cadavers or models, using a motion analysis system to measure patellar tracking, which may not fully replicate natural conditions. Our study focused on patients' radiological data and clinical outcomes, which hold substantial clinical relevance. In addition, the medial parapatellar approach in TKA is widely used, but this approach will unavoidably damage the vastus medialis muscle, leading to disruption of the peripatellar anatomy. We used the muscle‐sparing mini‐subvastus approach and utilized KA technology, without excessive soft tissue release, thereby better preserving the intrinsic constitution of the knee and achieving superior postoperative clinical outcomes.

The purpose of the study is: (i) to compare radiological parameters of patellar tracking and clinical outcomes following KA‐TKA and MA‐TKA procedures; (ii) to evaluate the clinical outcomes of LRR; (iii) to discuss more appropriate surgical strategies of TKA.

## 2 Methods

### 2.1 Sample Size Estimation

The sample size for this study was estimated based on the OKS, according to Winnock's study using PASS 15 software (Kaysville, Utah, USA). The estimation employed two‐sample *t*‐tests with the following parameters: Power = 0.9, Alpha = 0.05, number of groups = 2. The calculation indicated that an average group sample size of 40 would be sufficient to detect significant differences.

### 2.2 Inclusion and Exclusion Criteria

The inclusion criteria were as follows: (1) a definite diagnosis of knee osteoarthritis; (2) a varus knee; (3) the use of a cruciate‐retaining (CR) fixed plateau prosthesis (ATTUNE, DePuy Synthes, Johnson & Johnson). The exclusion criteria were as follows: (1) the presence of an extra‐articular deformity; (2) a prior patellar fracture or knee joint fracture; (3) a patella resurfacing during the operation; (4) a poor general condition and severe comorbidity; (5) incomplete postoperative imaging data or clinical follow‐up data.

### 2.3 Patient Selection

**FIGURE 1:**
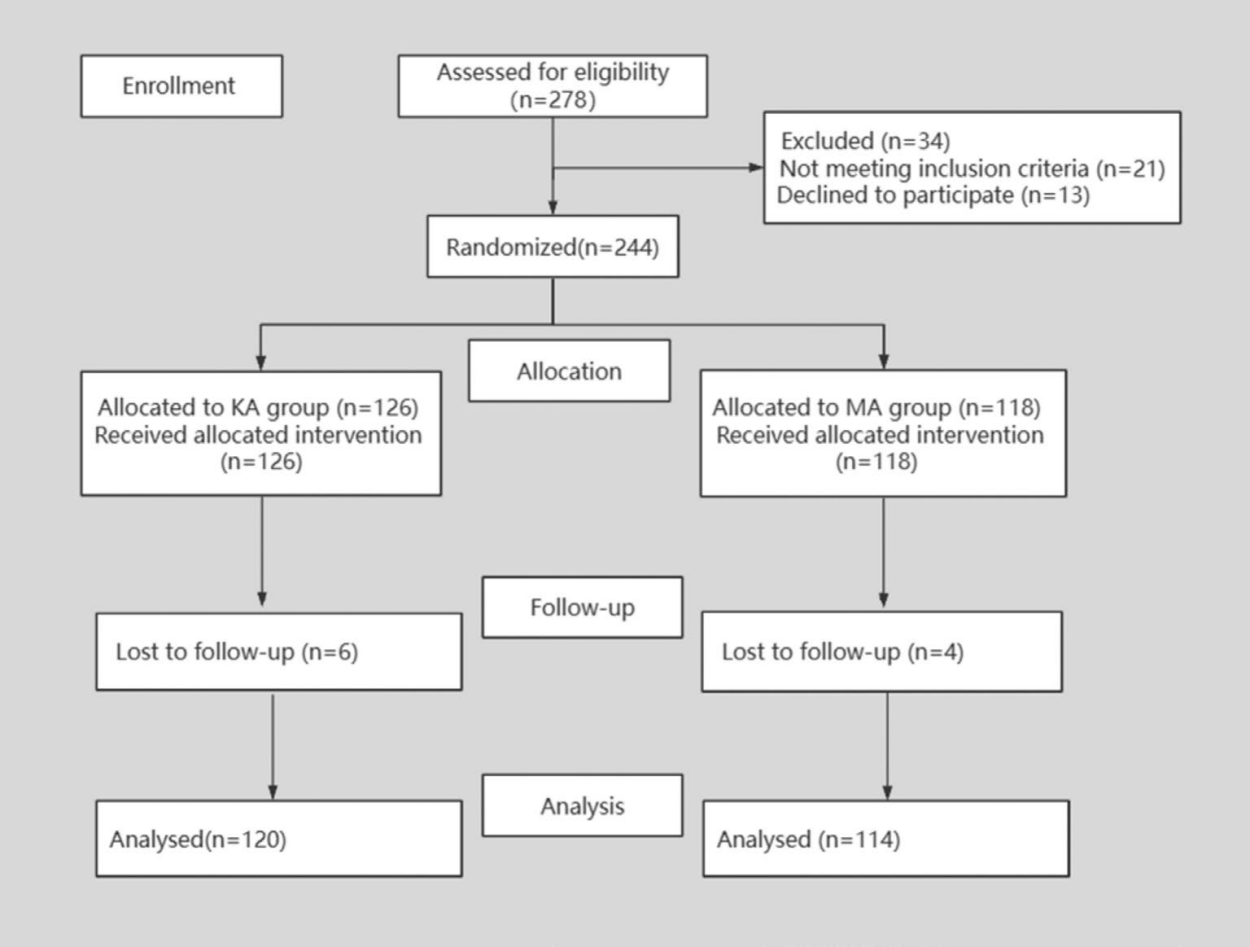
CONSORT flow diagram.

From January 2022 to October 2023, we conducted a prospective randomized controlled study on patients with knee osteoarthritis who underwent TKA. According to the inclusion criteria and exclusion criteria, 34 patients were excluded and 10 patients were lost to follow‐up, and a total of 234 patients were finally included in this study. SPSS 25.0 statistics software was used to group patients via random number allocation; 120 patients underwent KA‐TKA and 114 patients underwent MA‐TKA (Figure 1).

### 2.4 Ethical Approval

This study was approved by the Medical Ethics Committee of Fujian Provincial Hospital, China (No. K2021‐10‐004) and registered in the Chinese Clinical Trial Registry (ChiCTR2100054927) on 29/12/2021. Written informed consent was obtained from all the participants prior to the enrollment in this study.

### 2.5 Operative Procedure

**FIGURE 2:**
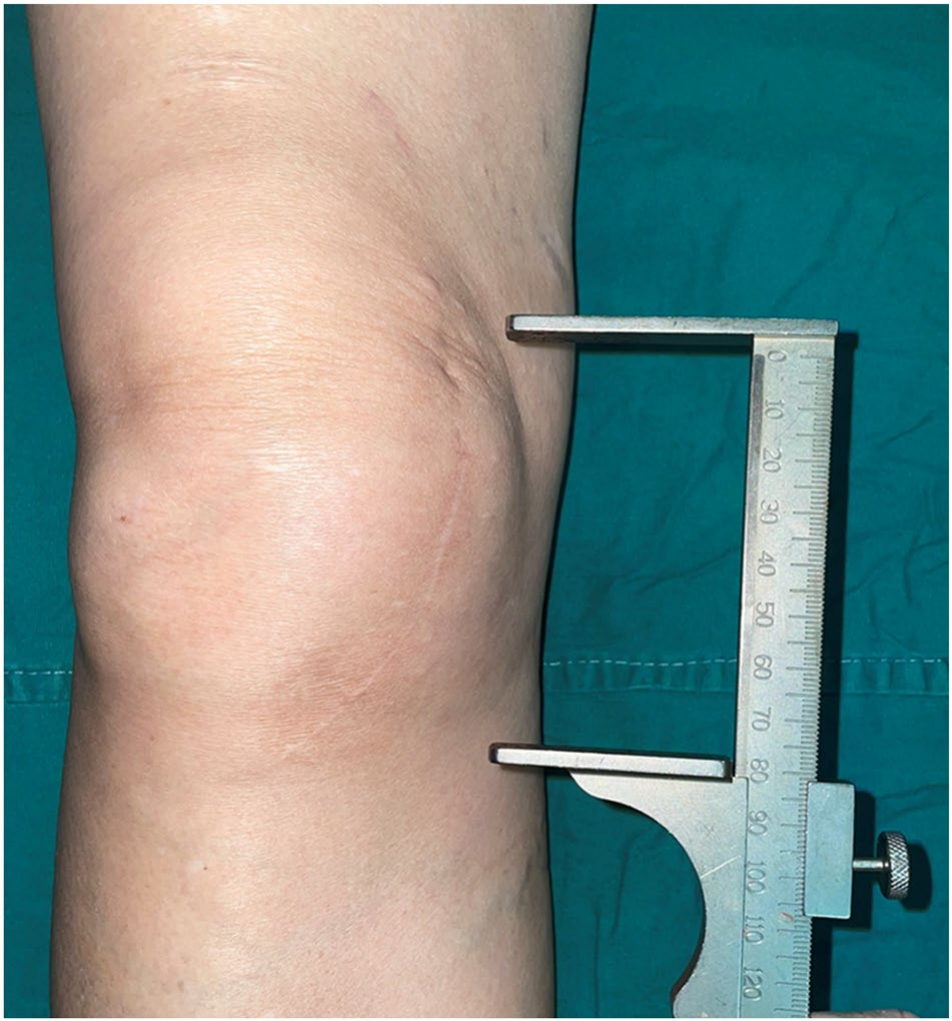
Postoperative scar of the mini‐subvastus approach.

All surgeries were performed by the chief surgeon with an assisting medical team that remained unchanged throughout the duration of this study. TKA was performed under general anesthesia, with the patient in the supine position, an upper tourniquet was applied to the affected limb (not used intraoperatively), and the incision was made via the mini‐subvastus approach (Figure 2). Further details of the TKA include nonvalgus patella, exposure of the knee joint, incision of the joint capsule, removal of the arthritic synovium and the anterior cruciate ligament, and preservation of the posterior cruciate ligament. The osteophytes of the medial condyle of the distal femur and the medial osteophyte of the tibial plateau were removed.

The surgical details of the MA group were as follows: for femoral intramedullary positioning, femoral condyle osteotomy was performed routinely at a valgus angle of 6°. The extramedullary positioning was performed on the tibial side; the mechanical axis of the tibia was perpendicular, and the plateau osteotomy was performed by tilting back 5°–7°. A gap block of appropriate thickness was placed. The posterior femoral condyle was externally rotated for osteotomy at 3°, and soft tissue was released according to the degree of tightness.

**FIGURE 3:**
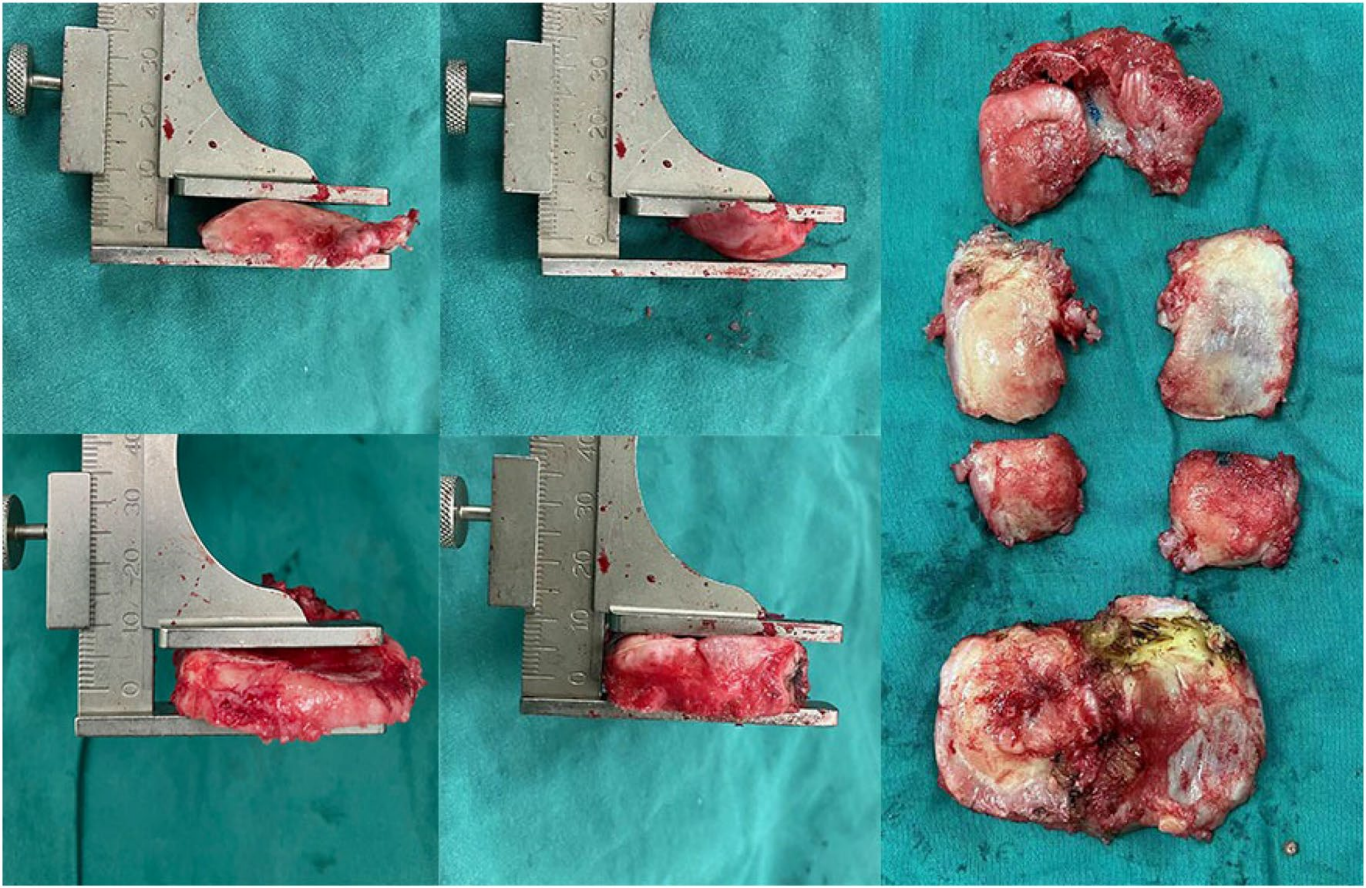
Use of calipers to measure the thickness of the resected bone.

**FIGURE 4:**
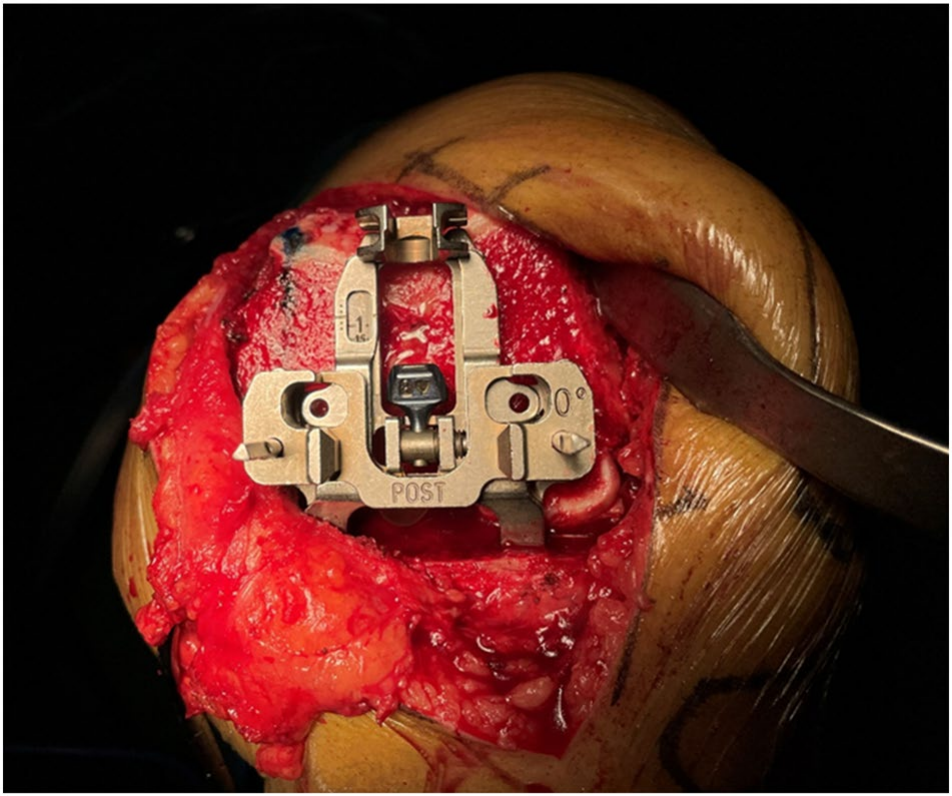
Osteotomy of the posterior femoral condyle at a 0° angle.

The surgical details of the KA group were as follows: femoral intramedullary position, valgus angle (the angle between the perpendicular line of the articular surface of the distal femur and the anatomical axis of the femur), osteotomy was performed according to the thickness of the prosthesis (osteotomy thickness + saw blade thickness + cartilage wear = prosthesis thickness), measurement with calipers after each osteotomy (Figure 3), and tibial varus and slope refer to the preoperative angle. The posterior femoral condyle underwent 0° osteotomy (Figure 4).

For both groups, denervated treatment of the patella was conducted, which involved removing the osteophytes, trimming the joint surface of the patella without patella resurfacing, using the “thumbless test” to test patellar tracking during the operation, and passively flexing and extending the knee joint to observe whether the patella was in the trochlear sulcus and dislocated outward. If the test was positive, the appropriate lateral patellar retinaculum was released. CR fixed‐plateau prostheses were used in this study.

### 2.6 Postoperative Management

Thirty minutes before the skin incision was made, cefuroxime sodium was administered intravenously. Postoperative antibiotics were not used, and multimodal analgesia and anticoagulant therapy were given. After waking from anesthesia and returning to the ward, the patient started straight leg raising, active and passive knee joint extension and flexion, and ankle pump training. Six hours postoperatively, the patient walked with the assistance of a walker. After the training, the knee was iced when lying in bed. The patient was allowed to go up and down stairs after 24 h and was finally discharged from the hospital when the lower extremity vascular color Doppler ultrasound was reexamined and no thrombus was detected. The therapist guides functional exercise after discharge.

### 2.7 Follow‐Up and Evaluation Indices

**FIGURE 5:**
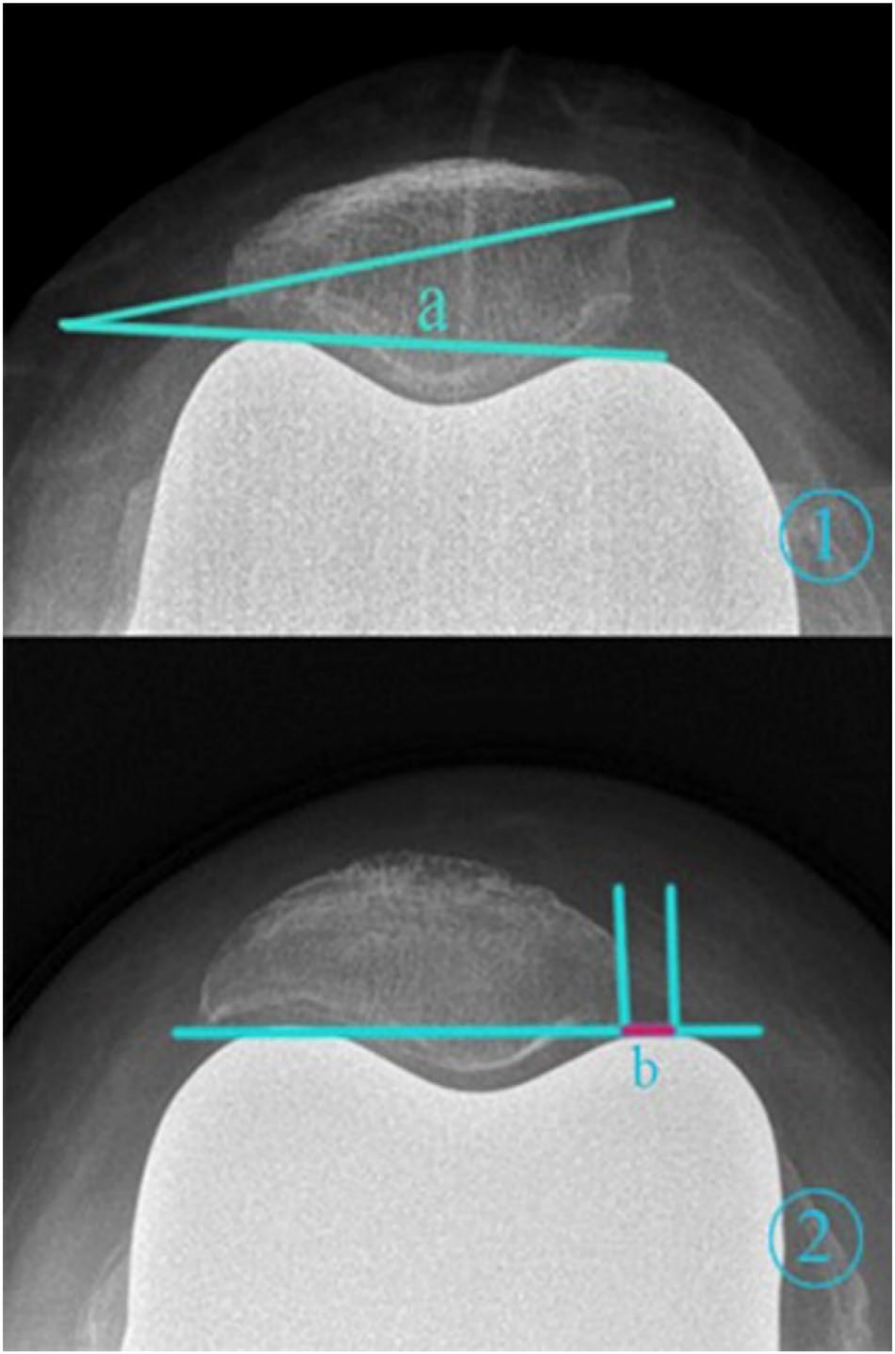
Patellar axial radiological images showing the patellar tilt and lateral patellar shift. (1) Patellar tilt is expressed as the angle (a) formed by the line connecting the highest points of the medial and lateral femoral condyles and the long axis of the patella. (2) The lateral patellar shift is expressed as (b). A line was drawn connecting the highest points of the medial and lateral femoral condyles; (b) is the distance between the two intersections of the perpendicular line drawn from the highest point of the medial femoral condyle and the innermost edge of the patella.

**FIGURE 6:**
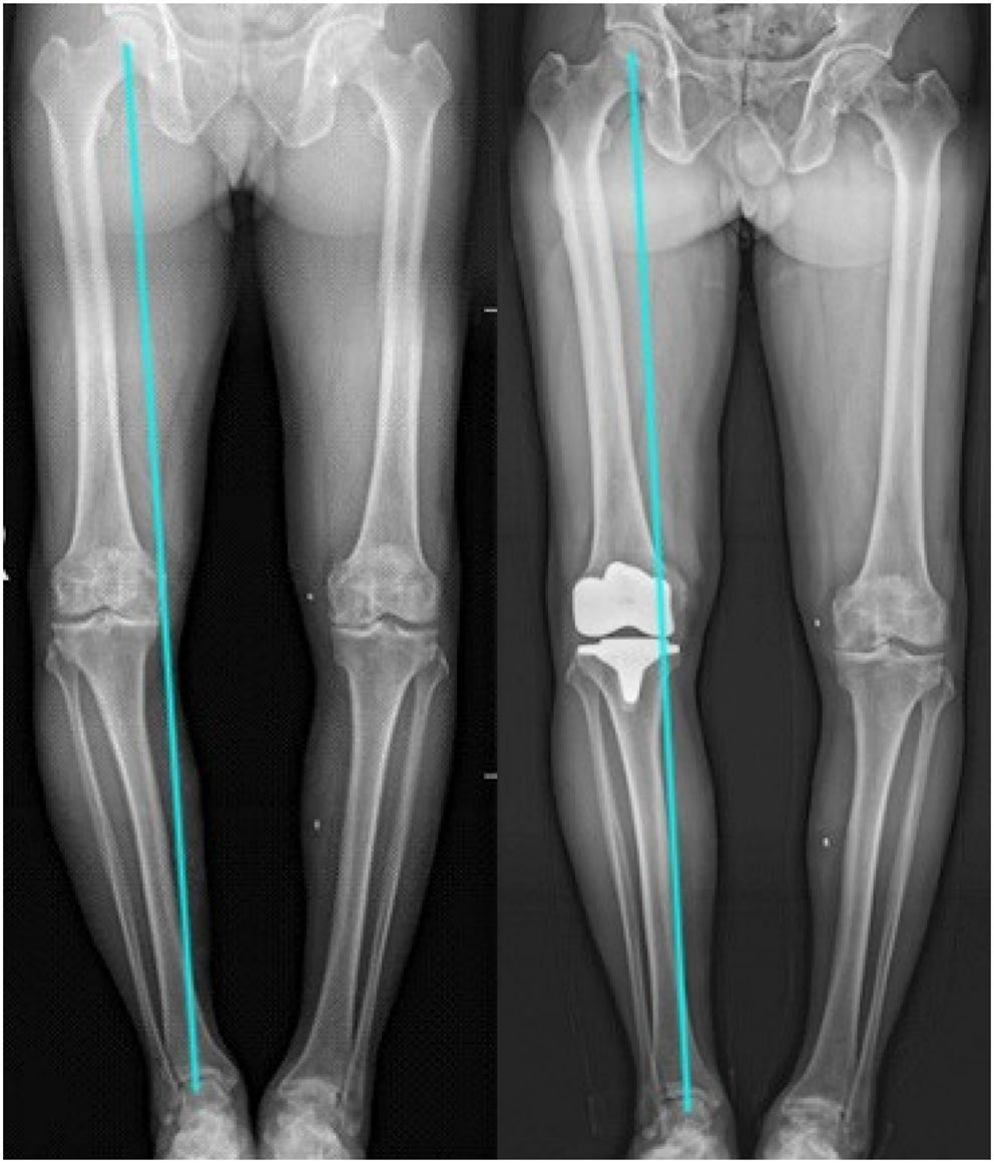
Postoperative long‐leg radiographs showing that KA‐TKA restored constitutional varus alignment. KA‐TKA, kinematic alignment‐total knee arthroplasty.

**FIGURE 7:**
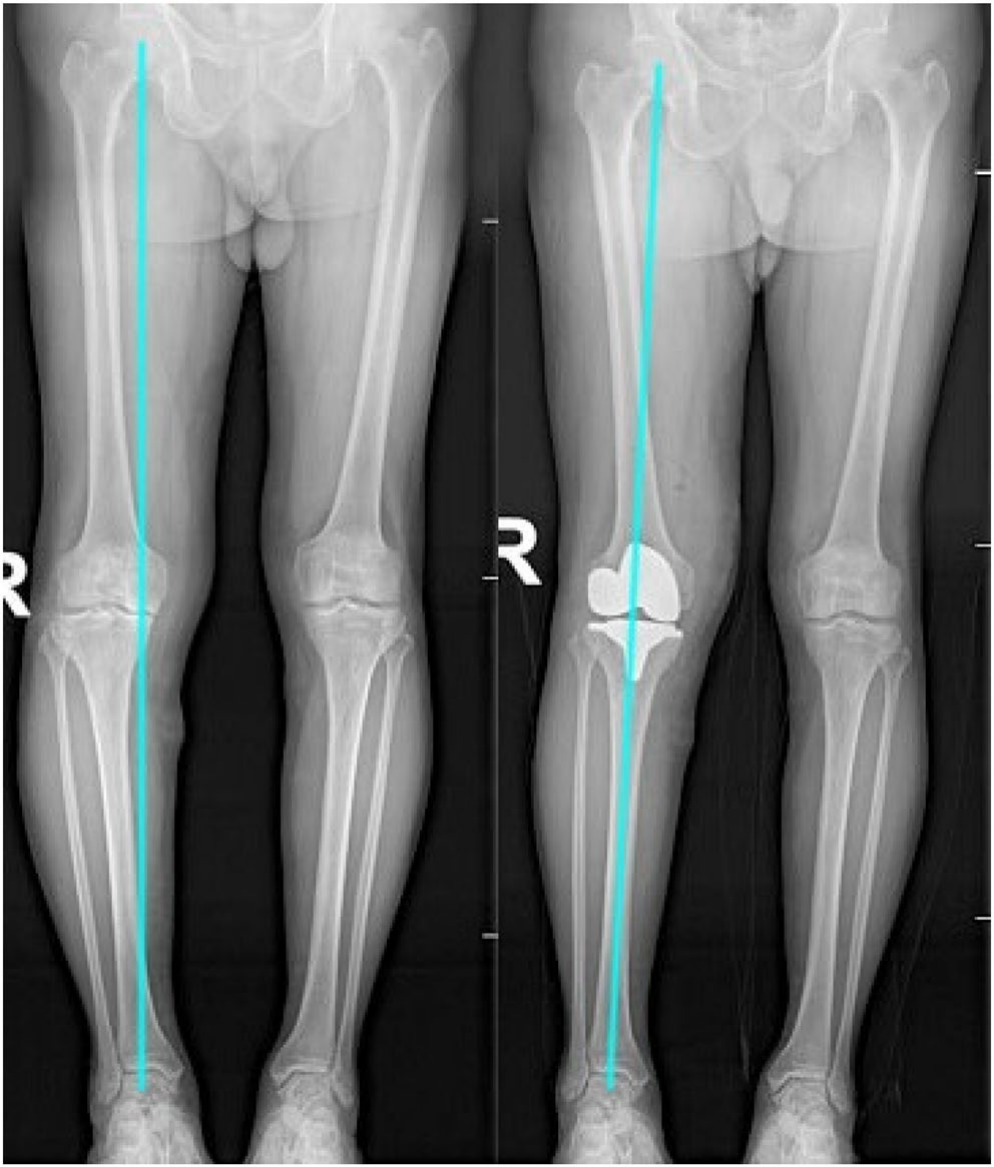
Postoperative long‐leg radiographs show that MA‐TKA changed the constitutional varus alignment to neutral. MA‐TKA, mechanical alignment‐total knee arthroplasty.

Regular postoperative outpatient visits were made in the 1st, 3rd, 6th, and 12th months, and the anteroposterior and lateral knee radiographs, weight‐bearing patellar axial radiographs with the knee flexed at 30° (Figure 5), and long‐leg radiographs of both lower limbs were reexamined (Figures 6 and 7) to check for complications, such as wound infection, patellar dislocation and fracture, osteolysis, and loose prosthesis. The patellar tilt and shift, preoperative and postoperative knee society score (KSS), oxford knee score(OKS), and intraoperative lateral retinacular release (LRR) rate were compared between the KA and MA groups (Table 2). Furthermore, data similar to those described above were compared between the LRR and non‐LRR groups (Table 3).

### 2.8 Statistical Methods

All statistical analyses were performed using SPSS version 25.0 (IBM Corporation, Armonk, NY, USA). The normality of the data were assessed, and the results indicated that the data were approximately normally distributed. Continuous variables were expressed as means ± standard deviation (SD); independent samples *t*‐tests were used to compare the differences in radiological parameters and clinical outcomes between groups. In addition, the LRR rate was compared using the chi‐square test. *p* < 0.05 was considered significant.

## 3 Results

### 3.1 Patient Baseline Characteristics

A total of 234 patients were included in the study; 120 patients underwent KA‐TKA, and 114 patients underwent MA‐TKA. There were no significant differences between the KA and MA groups regarding age, sex, and BMI (Table 1).

**TABLE 1:** Demographics of patients in the KA and MA groups.

Demographics	KA group	MA group	*p* value
Total	120	114	—
Age (years)	70.70 ± 7.32	70.26 ± 6.86	0.757
Sex			
Male	34 (28.3%)	25 (21.9%)	—
Female	86 (71.7%)	89 (78.1%)	—
Body mass index (kg/m^2^)	27.65 ± 2.81	27.01 ± 3.70	0.134

Abbreviations: KA, kinematic alignment; MA, mechanical alignment.

### 3.2 Radiological Parameters and Clinical Outcomes: KA Versus MA Group

No significant differences were observed in terms of pre‐ or post‐operative radiological parameters, preoperative functional scores between groups (*p* > 0.05) (Table 2). The postoperative patellar tilt and lateral patellar shift were both lower than they were before the operation. At 12 months postoperatively, the OKS and KSS of the KA group were higher than those of the MA group (*p* < 0.05) (Table 2). The LRR rate in the KA group was lower than that in the MA group (6.7% vs. 25.4%, *x* ^2^ = 15.476, *p* < 0.001).

**TABLE 2:** Preoperative and postoperative radiological parameters of patellar tracking and clinical outcomes between the KA group and the MA group.

	KA group	MA group	*p* value
	(*n* = 120)	(*n* = 114)	
Patellar tilt (°)			
Preop	12.18 ± 2.19	12.46 ± 2.85	0.593
Postop 12 months	10.12 ± 1.39	10.58 ± 1.92	0.176
Patellar shift (mm)			
Preop	2.68 ± 0.75	2.93 ± 0.70	0.088
Postop 12 months	2.22 ± 0.62	2.39 ± 0.77	0.229
OKS			
Preop	17.74 ± 1.64	18.15 ± 1.85	0.245
Postop 12 months	36.71 ± 2.42	29.69 ± 2.68	< 0.001
KSS			
Preop	50.56 ± 2.29	50.09 ± 2.63	0.347
Postop 12 months	85.90 ± 3.27	83.36 ± 4.47	0.002

Abbreviations: KA, kinematic alignment; KSS, knee society score; MA, mechanical alignment; OKS, oxford knee score.

### 3.3 Radiological Parameters and Clinical Outcomes: LRR Versus Non‐LRR Group

The preoperative patella tilt and lateral patellar shift were greater in the LRR group than in the non‐LRR group (*p* < 0.001), but the postoperative OKS was lower (*p* < 0.05) (Table 3).

**TABLE 3:** Preoperative and postoperative radiological parameters of patellar tracking and clinical outcomes between the LRR and non‐LRR groups.

	LRR	Non‐LRR	*p* value
	(*n* = 37)	(*n* = 197)	
Patellar tilt (°)			
Preop	14.65 ± 1.24	11.84 ± 2.47	< 0.001
Postop 12 months	11.20 ± 1.50	10.27 ± 1.68	0.058
Patellar shift (mm)			
Preop	3.44 ± 0.57	2.68 ± 0.70	< 0.001
Postop 12 months	2.36 ± 0.59	2.30 ± 0.70	0.728
OKS			
Preop	17.30 ± 2.03	18.08 ± 1.68	0.097
Postop 12 months	30.31 ± 3.73	33.79 ± 4.24	0.002
KSS			
Preop	49.94 ± 2.38	50.40 ± 2.49	0.488
Postop 12 months	83.92 ± 3.97	84.77 ± 4.13	0.435

Abbreviations: KSS, knee society score; LRR, lateral retinacular release; OKS, oxford knee score.

### 3.4 Complications of the Patients

During follow‐up, none of the patients experienced complications such as infection, patellar dislocation, fracture, osteolysis, or prosthetic loosening.

## 4 Discussion

### 4.1 Major Findings

The most important finding of our study is that KA‐TKA via the mini‐subvastus approach can achieve good patellar tracking. In addition, the OKS and KSS in the KA group were significantly higher than those in the MA group, and the intraoperative LRR rate was lower.

### 4.2 The Primary Distinction Between KA and MA

At present, there are still no guidelines that are universally accepted among surgeons for the selection of the optimal alignment strategy for TKA. However, most orthopedic surgeons have reached a consensus that MA is currently the most stable and reliable technique and the best guide in alignment selection [5]. The MA technique pursues neutral alignment; the prosthesis is vertical to the mechanical axis of the lower limbs, and the pressure of the lower limbs is evenly distributed in the inner and outer compartments of the tibia, thereby slowing the wearing down of bone cement and reducing the loosening of the prosthesis [6]. MA has been termed a biomechanical‐friendly approach, a mature and systematically applied technique [7, 8, 9, 10]. Nevertheless, the MA technique changes the intrinsic joint line of the patient, the alignment, the soft tissue balance, the Q angle, and the postoperative satisfaction of patients [11, 12, 13, 14, 15, 16]. KA is different from MA in that it restores the physiological and anatomical constitution of the knee. The key concept of KA is pure bone resection with negligible soft tissue release [17, 18, 19, 20]. Studies reported that the postoperative clinical outcomes of KA‐TKA in short‐term follow‐up were better than those of MA‐TKA through a systematic review and that the joint line orientation angle was more parallel to the ground and close to the native state of the knee in the patient [6, 21, 22]. Studies [23] suggested that, compared with MA‐TKA, the gait of patients after KA‐TKA is closer to the healthy state. Elbuluk et al. [24] proposed that the visual analog score (VAS) of KA‐TKA was lower than that of MA‐TKA at 6 weeks postsurgery, and the forgotten joint scores of KA‐TKA were also higher than those of MA‐TKA at 1–2 years after the operation. In this study, the postoperative OKS and KSS of patients in the KA group were greater than those in the MA group. On the basis of our present data, we suggest that postoperative functional exercise can be carried out early to promote rapid recovery.

### 4.3 The Mini‐Subvastus Approach Offers Significant Benefits

Most orthopedic surgeons use the standard medial parapatellar approach in TKA because it provides good intraoperative vision, but it will inevitably damage the medial vastus muscle, destroy the stable structure around the patella, decrease the knee extensor mechanism, and affect patient satisfaction and early postoperative recovery. Koh et al. [25] reported an increase in the lateral patellar tilt angle in the KA group after the medial parapatellar approach TKA relative to the MA group. Lozano et al. [26] proposed that the position of the femoral prosthesis used in the KA technique is more medial and allows internal rotation than that used in the MA technique and that this rotation is not conducive to patellar articulation and the femoral trochlea, resulting in an increase in the patellar lateral tilt. Notably, the researchers cited above performed TKA through the medial parapatellar approach, and most studies reached the same conclusion (the patellar tilt angle was larger in the KA group than in the MA group), whereas different conclusions were reached in this study, namely, that there was no significant difference in the patellar tilt between the mini‐subvastus approach KA‐TKA and MA‐TKA groups, indicating that the intact medial vastus muscle provides a stable mechanical environment for the patella that can effectively fight against the lateral femoral muscle and maintain the patella from dislocation to the outside and does not cause patellar tilt. Postoperative rehabilitation exercises were initiated within a short time in our study, which can reduce the incidence of deep venous thrombosis (DVT) [27]. Li et al. [28] noted that the joint range of motion, KSS, and hospitalization time of TKA patients who underwent the mini‐subvastus approach were better than those of patients in the medial parapatellar approach group at 6 months postoperatively. Bestorck et al. [29] reported that patients who underwent TKA through the mini‐subvastus approach had more advantages in various early postoperative evaluations. Wu et al. [30] conducted a meta‐analysis of 14 randomized controlled trials involving 1172 patients and concluded that the recovery time of straight leg raising after the mini‐subvastus approach was significantly shorter than that observed with the medial parapatellar approach.

### 4.4 Analyze the Reason for LRR and Evaluate Its Clinical Outcomes

**FIGURE 8:**
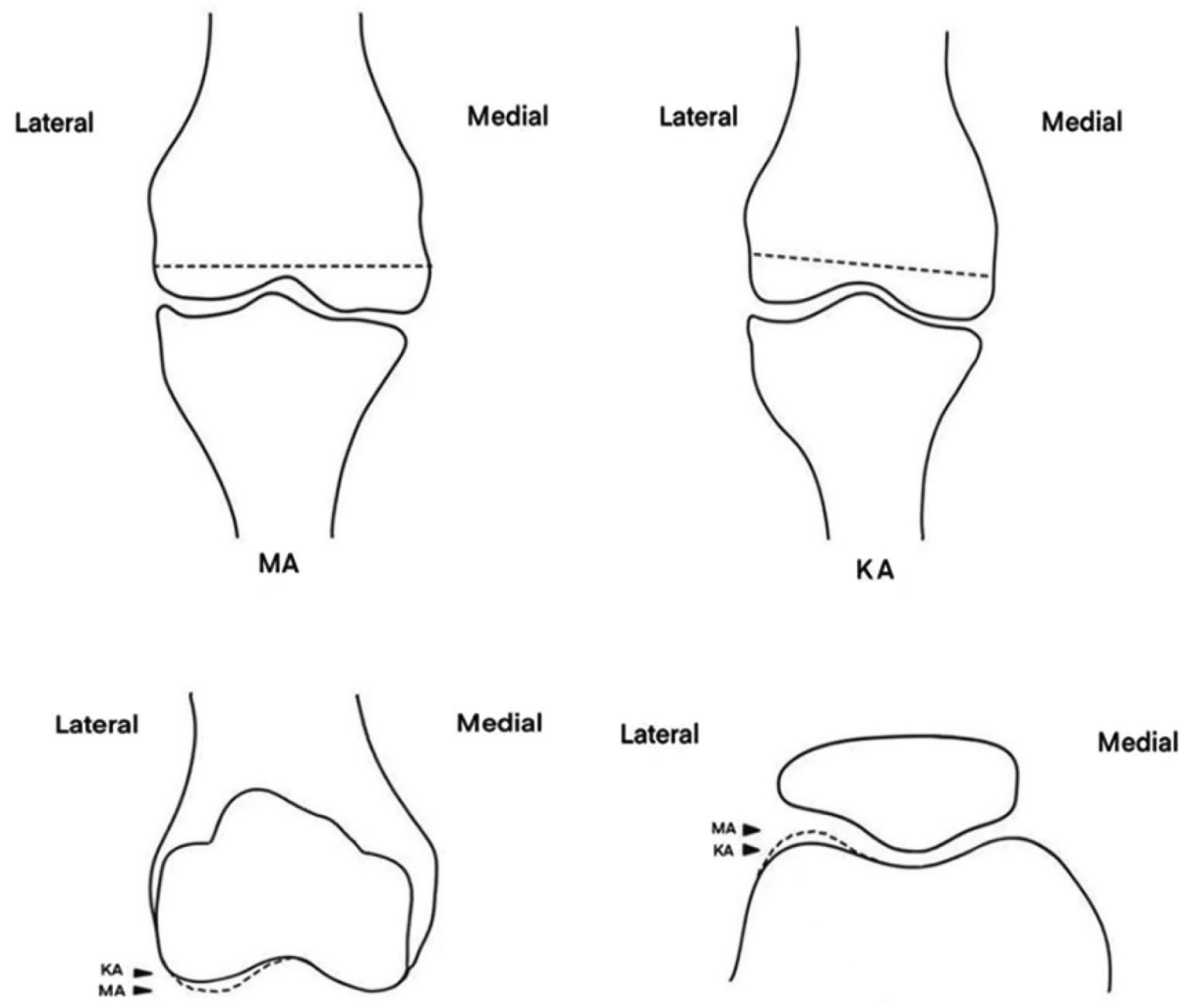
In KA‐TKA, equal bone resection from the medial and lateral femoral condyles is performed, which helps maintain the native joint line after prosthesis implantation. In contrast, MA‐TKA typically requires unequal resection of the femoral condyles, which often results in distalization of the joint line after prosthesis placement. As a result, patellofemoral overstuffing is more likely to occur during knee flexion in MA‐TKA.

The distal femur is the constitutional valgus in normal people, whereas the proportion of distal femoral valgus in patients with knee varus osteoarthritis is greater [31]. In MA‐TKA, more bone from the distal medial femoral condyle than from the lateral femoral condyle is removed, and the prosthesis is perpendicular to the mechanical axis of the lower extremity, changing the original joint line and resulting in the lateral condyle position of the femoral prosthesis moving downward relative to the preoperative period. Rivière et al. [32] reported that the average size of postoperative femoral prostheses after MA‐TKA was 0.6 mm and 1.25 mm greater than that of the outermost and distal condyle of the native lateral femoral condyle. When the knee is flexed, the distance between the patella and the lateral femoral condyle is narrower than usual, resulting in patellofemoral overstuffing (Figure 8), and the tension of the lateral patellar retinaculum increases and needs to be appropriately released. Harvey et al. [33] reported that the femoral valgus angle in the Chinese population was greater than that in Caucasians (1.35° for males and 2.01° for females), the postoperative lateral femoral condyle descending distance was greater, and the incidence of patellofemoral overstuffing was greater among Chinese individuals. The prosthesis on the market is designed on the basis of the anatomical details of the Caucasian population, which may not be suitable for Chinese. In this study, the LRR rate in the KA group was lower than that in the MA group, which was due to equal osteotomy of the distal femur in the KA group. The prosthesisis anatomically congruent with natural structure, retained the constitutional valgus in the coronal position, and did not change the joint line, whereas the patellofemoral overstuffing incidence decreased. Since MA‐TKA possibly leads to patellofemoral overstuffing, the question of “why so many patients (85/114) did not need LRR?” arises. We believe the reasons are as follows: First, an intact medial vastus muscle provides a stable mechanical environment for the patella, which prevents lateral shifting of the patella. Second, there was a lack of severe preoperative patella tilt and lateral patellar shift. Finally, management of the patellar articular surface can reduce patellofemoral pressure [34].

Another interesting finding of this study was that in the LRR group, the preoperative patella tilt and lateral patellar shift were greater (*p* < 0.001) and the postoperative OKS was lower (*p* < 0.05) than those in the non‐LRR group. This finding indicated that avoiding excessive soft tissue release contributes to better patient satisfaction. Patients with greater degrees of preoperative patella tilt and shift imply a greater chance of LRR [35].

### 4.5 Strengths and Limitations

Currently, most studies about patellar tracking after KA‐TKA are based on cadavers and models. Our study focuses on patients, which holds greater clinical significance. Unlike the traditional medial parapatellar approach, we used the muscle‐sparing mini‐subvastus approach and utilized KA technology, without excessive soft tissue release, thereby better preserving the intrinsic constitution of the knee and achieving superior postoperative clinical outcomes.

This study has several limitations. First, the follow‐up duration was insufficient. Second, this was a single‐center study, which may lead to confounding factors and bias. Third, the majority of the study participants were women, and the study outcomes may be influenced by sex bias. Fourth, postoperative weight‐bearing patellar axial radiographs only offer visualization at a single knee flexion angle (30°). Fifth, our study does not include an assessment of dynamic closed‐chain patella tracking. Finally, a single brand of prosthesis specifically designed for MA was used. In the future, multicenter, large‐sample, long‐term follow‐up studies are needed to further examine the effects of TKA with different alignment methods on patellar tracking.

## 5 Conclusions

In conclusion, our study showed that KA‐TKA via the mini‐subvastus approach can achieve both good patellar tracking and clinical outcomes. Avoiding muscle damage and refraining from excessive soft tissue release are crucial to improving postoperative patient comfort. In our opinion, KA‐TKA via the mini‐subvastus approach may be a more suitable surgical option.

## Author Contributions

B.S., Y.X., and G.W. conceived the idea for the study; they contributed equally to this work and should be regarded as co‐first authors. J.X. and Y.L. designed the study. F.L. and G.Y. collected the relevant data and followed the patients. L.C. and G.W. prepared the figures and tables. B.S. performed the statistical analyses. J.X. revised the manuscript. All authors listed meet the authorship criteria according to the latest guidelines of the International Committee of Medical Journal Editors and are in agreement with the manuscript.

## Ethics Statement

This study was approved by the Medical Ethics Committee of Fujian Provincial Hospital, China (No. K2021‐10‐004), and registered in the Chinese Clinical Trial Registry (ChiCTR2100054927) on 29/12/2021. Written informed consent was obtained from all the participants prior to the enrollment of this study.

## Consent

The authors have nothing to report.

## Conflicts of Interest

The authors declare no conflicts of interest.

## Data Availability

The data that support the findings of this study are available from the corresponding author upon reasonable request.
